# Police discretion in encounters with people who use drugs: operationalizing the theory of planned behavior

**DOI:** 10.1186/s12954-021-00583-4

**Published:** 2021-12-16

**Authors:** Brandon del Pozo, Emily Sightes, Jeremiah Goulka, Brad Ray, Claire A. Wood, Saad Siddiqui, Leo A. Beletsky

**Affiliations:** 1 The Miriam Hospital/Warren Alpert Medical School of Brown University, 164 Summit Avenue, Providence, RI 02906 USA; 2 Center for Behavioral Health and Justice, School of Social Work, Wayne State University, Detroit, USA; 3 Health in Justice Action Lab, Northeastern University, Boston, USA; 4 Missouri Institute of Mental Health, University of Missouri St Louis, St. Louis, USA; 5 School of Law and Bouve College of Health Sciences, Northeastern University, Boston, USA

**Keywords:** Police, Law enforcement, Overdose, Stigma, Opioids, Harm reduction, Naloxone, Syringes, Theory of Planned Behavior

## Abstract

**Background:**

Policing shapes the health risks of people who use drugs (PWUD), but little is understood about interventions that can align officer practices with PWUD health. This study deploys the Theory of Planned Behavior (TPB) to understand what influences police intentions to make discretionary referrals to treatment and harm reduction resources rather than arrest on less serious charges.

**Methods:**

On-line surveys integrating TPB constructs and adapting an instrument measuring police intentions to make mental health treatment referrals were completed by police employees in Indiana, Massachusetts, and Missouri. They also included items about stigma towards PWUD and attitudes and beliefs about opioid addiction, treatment, and recovery.

**Findings:**

Across the sites, 259 respondents perceived control over their decision to arrest for misdemeanors (69%) and confiscate items such as syringes (56%). Beliefs about others’ approval of referrals to treatment, its ability to reduce future arrests, and to increase trust in police were associated with stated practices of nonarrest for drug and possession and making referrals (*p* ≤ .001), and nonarrest for syringe possession (*p* ≤ .05). Stigma a towards PWUD was negatively associated with stated practices of nonarrest (*p* ≤ .05). Respondents identified supervisors as having the most influence over use of discretion, seriousness of the offense as the most influential value, and attitude of the suspect as the most important situational factor. The 17 Likert scale items analyzed had a Cronbach’s alpha of 0.81.

**Conclusion:**

The TPB offers untapped potential to better understand and modify police practices. In designing interventions to improve the health outcomes of police encounters with PWUD, further research should validate instruments that measure the relationship between these variables and discretionary intentions, and that measure role-relevant police stigma towards PWUD.

## Introduction

Fatal drug overdoses in the United States are at their highest recorded levels [28, 40]. Criminal-legal systems provide touchpoints with people with opioid use disorder (OUD) and afford opportunities to offer treatment and harm reduction measures to reduce overdose risk [32]. Police have frequent contact with people who use drugs (PWUD), but contribute to their health-risk environment through pathways, such as syringe and naloxone confiscation, and physical and verbal harassment [23]. These and other deleterious interventions can lead to syringe sharing, rushed injection, isolation while using drugs, and other risk behaviors [3]. Given the disproportionate burden of police interactions on minoritized and marginalized people, disparities in police enforcement can also translate into health disparities [26].

Encounters with police could instead be used to provide opportunities for harm reduction. Police officers could support diversion programs that offer treatment as an alternative to arrest and prosecution [51] and assert the value of carrying naloxone (the opioid overdose reversal medication) [48, 57]. Studies show officers acknowledge the public health benefits of access to sterile syringes, leaving them inclined to support efforts to decriminalize their possession [14]. Improving the outcomes of police encounters with PWUD requires resolving these conflicting dispositions in favor of health and away from punitive enforcement. One approach involves reforming the laws that empower police to act in the first place [15]. For example, some jurisdictions categorically decline to arrest people for possession of unprescribed addiction treatment medication [17] while the state of Oregon has decriminalized illicit drug possession altogether [33]. Another approach involves reshaping police officers’ knowledge, attitudes and beliefs [16]. Educating police has proven critical to the success of local syringe service programs [5, 53, 59] and Good Samaritan laws [9]. However, some studies found that evidence-based training can exacerbate younger officers’ negative attitudes towards PWUD [62] and harm reduction training for police should account for gender differences in behavior; male officers were found to be more likely to confiscate syringes from PWUD than female officers [37, 46, 50].

Regardless of the approach, improving health outcomes of police encounters with PWUD requires understanding how police use their discretion, and what motivates their intentions and behaviors. Individual officers have considerable discretion over non-felony arrests and enforcement (i.e., misdemeanors and violations) and harm reductionists rely on police to exercise it effectively. Officers can make an arrest or issue a citation, but as inevitable “social workers of last resort” [60], their power of discretion permits them to refer a person to addiction treatment, take them to a harm reduction facility, or issue naloxone and a warning, and doing these things in lieu of arrest. For example, the Ontario HIV Treatment network recommends that “rather than arresting people who inject drugs or confiscating injection equipment, law enforcement officials are encouraged to use discretion and refer individuals to appropriate community resources” [36].

Without guiding police use of direction by effectively shifting norms, expectations, training and policies, however, its undirected use can also create a gap between the intent of decriminalization and outcomes in practice when officers make arrests for ancillary charges. For example, in some states, legalizing syringe possession doesn’t prevent charging for the drug residue in a used one [8]. Some jurisdictions report that despite police policy changes emphasizing referrals to treatment, they have not seen a downstream change in police behavior towards PWUD [6]. Ideally, officers would refer people with OUD to medications for addiction treatment (MAT)^1^ or direct them towards harm reduction resources as an alternative to arrest, yet this is often not the case. Evidence suggests race skews police perceptions of danger where officers perceive Black suspects as more formidable and dangerous than whites [61]. This may incline officers to exert physical control over a situation rather than make a referral.

The effective use of discretion hinges on a combination of knowledge and the right behavioral intentions, but police discretion remains under-studied and difficult to influence [34]. A scoping review of police harm reduction training concluded that “the available literature contains significant gaps pertaining to descriptions of training development, design and content specific to facilitating positive police-PWUD interactions” [30]. To fill these gaps, this study assess whether the Theory of Planned Behavior (TPB) [1] can be employed to better understand what factors influence police discretionary intentions in drug enforcement, and offers evidence as to what approaches can promote decisions that improve the health outcomes of encounters with PWUD.

**Fig. 1:**
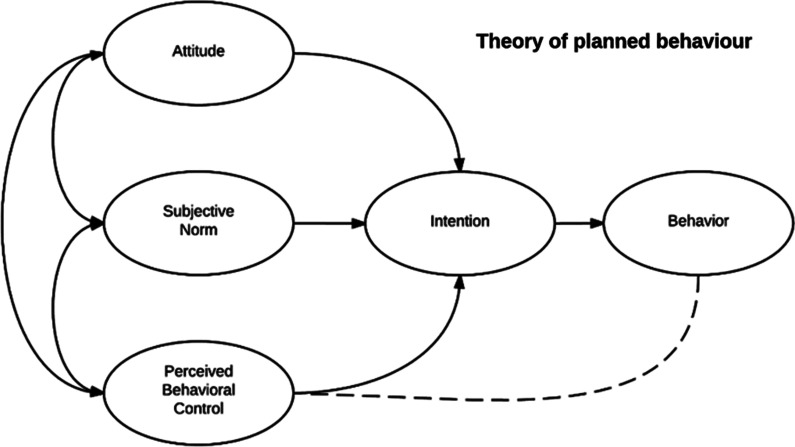
The theory of planned behaviour, Azjen (1991) (creative commons license)

The TPB is based on the premise that a person’s intentions can reliably predict their behavior. These behavioral intentions are formed by the interplay of three variables: perceived behavioral control, subjective norms, and attitudes about the behavior (Fig. 1). Perceived behavioral control is expressed as the strength of a person’s belief that they are able to carry out the behavior in question. Subjective norms are the expectations and pressures that regulate behaviors in response to other people’s reactions to them, while attitudes are personal dispositions that shape a person’s view of a behavior and their desire to undertake it. This study surveys police officers to better understand how to operationalize these variables, and learn what their initial dispositions are when considering the use of discretion towards people who use drugs.

The TPB is an adaptable theory that has been used to explain intentions to exercise [18], wear seatbelts [54], commit traffic infractions [45], bicycle with a helmet [31], quit smoking [43], and use illicit drugs [2]. Its variables have been explicitly operationalized for policing studies only a handful of times, however [25, 29, 44]. A study by Ishoy [27] conducted qualitative interviews aimed at operationalizing the theory’s three variables to explain how police make discretionary enforcement decisions. Findings revealed that officers believe they have control over their enforcement actions in a wide range of circumstances and their attitudes strongly influence decisions about minor infractions. Their perceived seriousness of the offense also figured heavily into formulating their intentions. A second study assessed whether police officers’ intentions to refer a person to psychiatric treatment in lieu of arrest were stronger if the officer had received Crisis Intervention Team (CIT) training [11]. It concluded that officers with the training had more confidence in their ability to make referrals and a more positive attitude towards the referrals.

For the current study, we developed survey items based on the foundational work of Ishoy [27] and adapted from the work of Compton et al. [11]. We explored if the constructs of the TPB can be suitably operationalized to examine the effects of perceived behavioral control, subjective norms, and attitudes on police behavioral intentions about drug enforcement and related discretionary behavior. The goal was not to validate the instrument used here, but to determine whether initial results make the case for developing and validating instruments tailored to measure police-specific stigma and behavioral intentions during encounters with PWUD. Our analysis applies this well-established theory to a new line of inquiry, producing data that can be used for future analyses on a larger scale and to help develop police harm reduction training, policies, and other interventions accordingly.

## Data and methods

Data were collected via online instruments administered in advance of Safety and Health Integration in the Enforcement of Laws on Drugs (SHIELD), a series of training sessions organized by the Indiana Law Enforcement Academy, the Missouri Drug Overdose Trust and Safety (DOTS) project, and the Massachusetts site of the Helping to End Addiction Long-term Communities Study [55]. The SHIELD Training Initiative is designed to improve occupational safety and preparedness of police and other emergency personnel for their role in overdose crisis response [13, 52]. The training curriculum frames effective SUD treatment, sterile syringe access, naloxone distribution, and other harm reduction interventions as not only benefiting public health, but decreasing officer fatigue and burnout, and lowering the probability of police contact with hepatitis and HIV [52]. Data were collected during virtual trainings between November 2020 and March 2021. In addition to collecting demographic data about respondents, the survey included items assessing knowledge, attitudes and beliefs about addiction, treatment, harm reduction, stigma, the COVID-19 pandemic, fentanyl exposure, and appropriate enforcement responses to drug use. It also contained a series of questions about officers’ ability to control the enforcement decisions they make and what factors influenced their intentions. This study was determined exempt by Northeastern University, Wayne State University, and University of Missouri St. Louis IRBs. Analyses were conducted using Stata [58].

Capitalizing on an opportunity to access police officers in a training environment across different jurisdictions, two overlapping instruments were used for this study. The principal one was administered to study participants in Indiana and Massachusetts to set a pre-class baseline. Two others containing a subset of the study’s questions of interest were administered in Missouri. The instruments were not identical because the particular evaluation goals differed somewhat across settings. Three hundred sixteen (316) participants started the surveys. The comprehensive one administered in Indiana and Massachusetts was completed in its entirety by 173 police employees, 30 from Massachusetts and 143 from across Indiana. The Missouri battery of selected questions was completed by 86 respondents from several agencies, 32 of whom were enrolled in police academy recruit training. In total, 259 respondents completed either the instrument or the shorter battery of questions (including responses with minor omissions not relevant to the analyses here), and 57 partial completions were excluded from the analysis using listwise deletion. This yielded a completion rate of 82% of the starts. Because the training was conducted via Zoom, we do not know how many officers never started the instrument, but a systematic review of police survey research placed the mean survey completion rate across all methods of administration at 64.3%, with a 79.4% response rate for in-person surveys [41, 42]. The method of administration approximated an in-person approach in a time of pandemic: all participants in the training were asked to complete their instruments by live Zoom instructors at the beginning of class, were assured anonymity, and were provided a clickable link that would work on phones, computers and tablets. Fifteen (15) minutes were set aside specifically for the completion of the survey, during which time instruction was paused. Instructors issued periodic reminders they would resume when the group finished their surveys.

Responses indicated the sample was representative of a typical cohort of officers, though it contained more females than are usually found in U.S. police departments (18% vs. 13%) [19], and their mean age was slightly older than the national mean age of a police officer (41.6 vs 39.5) (Data USA, 2020). Of the 259 respondents, 60% (*n* = 153) had an associate’s degree or greater, a majority were assigned to enforcement, investigative, and community outreach capacities, and 40% (*n* = 104) were early in their careers, with fewer than 8 years in policing. In terms of race, 86% (*n* = 222) indicated they were white, 9% (*n* = 24) indicated they were Black, and the remainder chose not to answer or selected other races.

## Results

### Perceived behavioral control over drug enforcement decisions

All respondents completed two statements about perceived control over enforcement decisions: one was “whether or not I arrest a suspect for a nonviolent misdemeanor or violation is…” and the other was similarly phrased about control over confiscating items such as syringes. Responses were measured on a 1–6 Likert scale, with two anchor points: 1 being “not under my control at all,” and 6 being “entirely under my control.” A majority of officers felt they had significant or total control over their personal decision to arrest a suspect, or confiscate items that could be considered drug-related contraband (Table 1). The strongest sentiments about control were for arrest: 35% (*n* = 90) of officers felt they had total control over whether they made an arrest for a nonviolent misdemeanor or violation (a score of 6), and 69% (*n* = 179) felt they had some amount of control (scores of 4–6). As for contraband, 31% of officers (79) felt confiscation was entirely under their control, and 56% felt they had some amount of control (*n* = 145). Very few officers felt they had no control at all over arrest or confiscation (16 and 38, respectively, or 6% and 13%). The bivariate correlation between control over arrests and control over confiscation was 0.498 (*p* ≤ 0.001).

**Table 1:** Officers’ perceived control over decisions to arrest and confiscate in drug-related encounters (1–6 Likert scale) (N = 259)

Enforcement type	Mean (SD)	No control (1)	Lack of control (1–3)	Some control (4–6)		Total Control (6)
Control over arrest	4.42 (1.56)	16 (6%)	79 (30%)	179 (69%)		90 (35%)
Control over confiscation	3.96 (1.79)	38 (13%)	114 (44%)	145 (56%)		79 (31%)

Reported discretionary behaviors concerning drug-related arrests and confiscation (1–6 Likert scale) (N = 173)						
Discretionary behavior	Mean (SD)	Always (1)		Inclined towards (1–3)	Inclined against (4–6)	Never (6)
Confiscate naloxone	5.50 (1.22)	6 (3%)		16 (9%)	157 (91%)	140 (81%)
Confiscate syringes	2.35 (1.72)	81 (47%)		139 (80%)	34 (20%)	22 (13%)
Not arrest for syringe poss	3.20 (1.75)	44 (25%)		108 (62%)	65 (38%)	27 (16%)
Not arrest for drug poss	3.87 (1.68)	21 (12%)		78 (45%)	95 (55%)	40 (23%)
Refer to treatment/nalox	2.94 (1.76)	44 (25%)		123 (71%)	50 (29%)	29 (17%)

To further gage perceived behavioral control, officers were asked how often they confiscate syringes and naloxone; use their discretion not to make arrests for possession of syringes and illicit drugs; and refer people to MAT or naloxone. Answer choices were on a 6-point Likert scale, with 1 being “always” and 6 being “never.” Respondents reported being inclined to use their discretion not to arrest someone for syringe possession 66% of the time, with 35% of officers saying they would always use their discretion not to arrest. When someone possesses illicit drugs, 48% reported they are inclined not to make an arrest for it, and 15% said they would always use their discretion not to arrest. However, confiscation of syringes was a different matter. Of the respondents, 77% were inclined to confiscate them, and 43% reported they would always confiscate. Only 23% were inclined against confiscation, and 15% said they would never confiscate (Table 3).

### Norms influencing discretionary drug enforcement

Normative factors that could influence an officer’s decision to make an arrest for a nonviolent misdemeanor were divided into three categories: beliefs about actions and consequences, meeting the expectations of others, and factors specific to the situation at hand. For each category, all respondents were asked to rank the factors from most to least influential when they “…have the discretion to make an arrest for a nonviolent misdemeanor or violation.” From four possible beliefs about actions and consequences, 72% (186) of officers reported the seriousness of the offense was most likely to influence their decision to arrest, a factor which was identified as highly influential in prior studies [27, 39, 56], followed by the availability of effective alternatives (Table 2a). Among the people who could influence the decisions of officers, supervisors wielded the greatest influence by a clear margin, followed by colleagues and peers, but overlapping confidence intervals prevent distinguishing the influence of the remaining groups (Table 2b).

**Table 2:** Norms and attitudes influencing discretionary drug enforcement (4 ranked choices) (N = 259)

Rank	Factor	Mean position (SD)	95% Confidence interval
**a. Normative values at work in making a discretionary arrest**			
1	Seriousness of the offense	1.38 (0.70)	1.30–1.47
2	If effective alternatives exist	2.65 (0.97)	2.53–2.77
3	The need for there to be consequences	2.97 (0.82)	2.87–3.07
4	Arrests should be made when laws are broken	3.00 (1.08)	2.86–3.13
**b. Influence of the expectations of others in making a discretionary arrest**			
1	Expectations of supervisor(s)	1.90 (0.82)	1.80–2.00
2	Expectations of colleagues/peers	2.61 (0.95)	2.50–2.73
3	Expectations of friends/family	2.69 (0.98)	2.54–2.84
4	Expectations of community	2.80 (1.18)	2.65–2.94
**c. Influence of situational factors in making a discretionary arrest**			
1	Attitude of the suspect	1.63 (0.71)	1.54–1.72
2	Personal sense of right and wrong	2.04 (1.02)	1.91–2.16
3	Suspect hasn’t learned their lesson yet	2.72 (0.84)	2.62–2.82
4	Personal factors (overtime/work schedule)	3.69 (0.69)	3.53–3.70

Ishoy [27] concluded specific situational factors influence an officer’s attitude about enforcement more than the general attitudes the officers held prior to assessing a situation. The data demonstrates this in one sense: the attitude of a suspect was rated to be most influential in deciding to make an arrest, with 49% (*n* = 126) of officers ranking it as most influential, and another 42% (*n* = 108) ranking it as second most, indicating 91% ranked it either first or second most important. This was followed by the officer’s personal sense of right and wrong, which was ranked most important by 41% (*n* = 105) of respondents (Table 2c).

Context for ranking the attitude of the suspect as the most influential of these four factors may be provided by officers’ views on addiction and stigma towards opioid users, as 65% (112) of the 173 who completed the more comprehensive instrument agreed that people who become addicted to opioids are “to blame for their own condition,” and 80% (138) agreed they “won’t hesitate to lie when it benefits their addiction” (Table 3). Of note is the last place ranking of personal factors, such as overtime and an officer’s work schedule when deciding to arrest. A trope of police culture is that officers’ intentions to enforce the law are based in part on the need to accrue arrest-related overtime or to avoid disrupting their work/life schedule. While research supports these influences [10, 38], the responses here appear to heavily discount them; they were ranked least influential by 71% (185) of officers, and second least by 21% (55). Prior research suggests the possibility of a social desirability response bias.

**Table 3:** Approval of treatment as an alternative to arrest and beliefs about addiction and treatment (1–6 Likert scale) (N = 173)

	Mean (SD)	Very likely (1)	Likely (1–3)	Unlikely (4–6)	Not at all likely (6)
Supervisors would approve of referrals	2.64 (1.65)	60 (35%)	130 (75%)	43 (25%)	18 (10%)
Coworkers would approve of referrals	2.67 (1.58)	50 (29%)	131 (76%)	42 (24%)	17 (10%)
Friends/neighbors would approve of referrals	2.67 (1.53)	49 (28%)	128 (74%)	45 (26%)	12 (7%)
Referrals to treatment reduce future arrests	2.83 (1.30)	29 (17%)	134 (77%)	39 (23%)	10 (6%)
Referrals to treatment increase trust in police	2.69 (1.32)	36 (21%)	139 (80%)	34 (20%)	8 (5%)

	Mean (SD)	Strongly agree (1)	Agree (1–3)	Disagree (4–6)	Strongly disagree (6)
People who become addicted to opioids are to blame for their own condition	3.22 (1.30)	15 (9%)	112 (65%)	61 (35%)	12 (7%)
People who are addicted to opioids won’t hesitate to lie when it benefits their addiction	2.35 (1.53)	66 (38%)	138 (80%)	35 (20%)	11 (6%)
I would worry about a person in recovery for opioid addiction taking care of my family’ s children for a few hours	2.66 (1.67)	65 (38%)	126 (73%)	47 (27%)	17 (10%)
People become addicted to opioids because they lack the willpower to stop before it’s too late	3.76 (1.55)	16 (9%)	81 (47%)	92 (53%)	29 (17%)
Opioid/heroin users will use more opioids/heroin if they know they have access to naloxone	3.44 (1.57)	22 (13%)	96 (55%)	77 (45%)	25 (14%)
Harm reduction services that distribute items such as syringes and naloxone condone a person’s addiction	3.83 (1.63)	18 (10%)	76 (44%)	97 (56%)	39 (23%)
There should be a limit on the number of times one person receives naloxone to reverse an overdose	4.39 (1.75)	16 (9%)	56 (32%)	117 (68%)	76 (44%)
Everyone at risk of experiencing or witnessing an overdose should be given a supply of naloxone	2.56 (1.54)	57 (33%)	135 (78%)	38 (22%)	12 (7%)
People can successfully overcome an opioid addiction	2.14 (1.23	65 (38%)	155 (90%)	17 (10%)	6 (3%)
An officer who completed treatment for addiction to prescription opioid pills could be trusted to return to duty	2.90 (1.22)	22 (13%)	127 (73%)	46 (27%)	4 (2%)

### Other beliefs and influences on police behavioral intent

Respondents were consistent in believing that supervisors, coworkers and friends/neighbors would approve of referrals to MAT rather than arresting people with OUD. For each of these variables, approximately three-fourths of the 173 respondents agreed these groups would approve of such referrals (Table 3). A greater majority felt that referrals to treatment would both reduce the number of future arrests, a belief with a basis in evidence [4, 7, 21, 24], and would increase a suspect’s trust in the police “since they are getting the help they need” (77% and 80%, respectively). The Cronbach’s alpha for the five variables was 0.89, and they all correlated with each other at *p* ≤ 0.001 (see Table 4), suggesting significant internal consistency as a measure of beliefs in the effectiveness of MAT and widespread support for it.

**Table 4:** Correlation matrix (1–6 Likert scale) (N = 173)

Variables	1	2	3	4	5	6	7	8	9
1	1								
2	.910**	1							
3	.697**	.752**	1						
4	.547**	.556**	.596**	1					
5	.484**	.480**	.544**	.725**	1				
6	−.122	−.086*	−.036	−.091	−.0788	1			
7	.080	−.119	−.071	−.078	−.026	.184*	1		
8	−.203*	−.222*	−.154*	−.138	−.077	.307**	.582**	1	
9	−.047	−.070	−.043	−.120	−.112	.500**	− 0.050	.027	1
10	−.100	−.070	−.058	−.077	−.126	.261**	− .036	.068	.327**
11	−.106	− .098	−.085	−.112	−.175*	.175*	.241*	.333**	.146
12	− .178*	− .222*	−.216*	−.261**	−.216*	.163*	.145	.272**	.238*
13	.453**	.386**	.345**	.423**	.353**	−.068	− .065	− .238*	− .062
14	.349**	.299*	.243*	.281**	.164*	−.108	− .166*	− .226*	− .072
15	.297**	.237*	.180*	.259**	.193*	−.106	− .186*	− .212*	.020
16	.168*	.227*	.194*	.206*	.203*	−.099	− .160*	− .272**	− .085
17	.118	.134	.116	.237*	.284**	−.116	.047	.043	− .120

Variables	10	11	12	13	14	15	16
1							
2							
3							
4							
5							
6							
7							
8							
9							
10	1						
11	.255**	1					
12	.298**	.396**	1				
13	− .054	− .183*	− .290**	1			
14	− .052	− .103	− .126	.252**	1		
15	− .156*	− .130	− .007	.010	.542**	1	
16	− .243*	− .191*	− .554**	.018	.017	.067	1
17	− .059	.015	− .050	− .0643	.064	.086	.125

*p ≤.05; **p ≤.001 Cronbach's alpha for all variables=0.81

1. My supervisor would approve of me referring a subject who appears to have an opioid addiction to MAT as an alternative to arrest

2. My coworkers would approve of me referring a subject who appears to have an opioid addiction to MAT as an alternative to arrest

3. My friends or neighbors would approve of me referring a subject who appears to have an opioid addiction to MAT as an alternative to arrest

4. Referring subjects who appears to have an opioid addiction to MAT helps reduce future arrests

5. Referring a subject who appears to have an opioid addiction to MAT increases his/her trust in the police, since they are getting the help they need

6. People who become addicted to opioids are to blame for their own condition

7. People who are addicted to opioids won’t hesitate to lie when it benefits their addiction

8. I would worry about a person in recovery for opioid addiction taking care of my family’s children for a few hours

9. When people become addicted to opioids, it’s because they lack the willpower to stop before it’s too late

10. Opioid/heroin users will use more Opioid/heroin if they know they have acesse to naloxone

11. Harm reduction services that distribute items such as syringes and naloxone condone a person’s addiction

12. There should be a limit on the number of times one person receives naloxone to reverse an overdose

13. How often do you provide information or make referrals to drug treatment or naloxone distribution programs?

14. When someone has illicit drugs, how often do you use your discretion not to arrest for drug possession?

15. When someone has a syringe, how often do you use your discretion not to arrest for syringe possession?

16. Everyone at risk of experiencing or witnessing an overdose should be given a supply of naloxone

17. People can successfully overcome an opioid addiction

Table 4 examines associations between 17 variables of interest in the survey. Among the strongest were an associated belief that PWUD would not hesitate to lie to benefit their addiction (*n* = 138; 80%) and worrying about a person in recovery from OUD taking care of an officer’s family’s children for a few hours (*n* = 136; 73%). The strengths of these two beliefs were closely correlated (*r* = 0.582, *p* ≤ 0.001), suggesting a relationship between perceived truthfulness and trust. These variables were also negatively associated in pairwise comparisons with not making arrests for drug and syringe possession (*p* ≤ 0.05), suggesting that police stigma towards PWUD, insofar as stigma suggests PWUD are by nature untruthful and untrustworthy, impedes the use of discretion. The four variables had a Cronbach’s alpha of 0.65. There were also statistically significant associations between beliefs that PWUD were to blame for their own condition, that addiction results from a lack of willpower, that harm reduction services condone PWUD’s behavior, and that naloxone distribution increases illicit opioid use.

In terms of the TPB, a relationship was observed between the five “widespread support and effectiveness” variables discussed above, and respondent disclosures of actual behaviors of making referrals and not making arrests for drug and syringe possession. The way these eight variables associate suggests a strong relationship between a beliefs in the effectiveness of MAT, support for referral to MAT among peers, the public and supervisors, and the actual practice of health-improving discretionary behaviors. All 15 pairwise comparisons yielded correlation coefficients significant at *p* ≤ 0.05, and 10 were significant at *p* ≤ 0.001. The 17 variables had an overall Cronbach’s alpha of 0.81, suggesting further research could examine the items’ suitability for development into validated instruments measuring both police stigma towards PWUD and behavioral intentions utilizing the constructs of the TPB.

## Discussion

This study measured police attitudes, beliefs, and intentions, revealing that they map onto the variables of the TPB variables and suggesting avenues for further study using the theory’s constructs. Officers perceived strong control over their discretionary behavior, acknowledged the presence of norms and attitudes that influenced their intentions, and asserted that some norms influenced them more strongly than others. The strong influence of a suspect’s attitude on police discretionary decisions found in this study aligns with research that perceived disrespect towards police by suspects is likely to be reciprocated [20, 49], and that a suspect’s demeanor inspires emotions in police officers that affect decisions [41, 42].

The perceived tendency of PWUD to lie may be considered by officers as disrespectful or obstructionist, fostering negative attitudes towards alternatives to arrest. It is notable that attitude was ranked most important of the situational factors by all groups of respondents except Missouri’s police academy recruits, who had yet to have substantive professional contact with PWUD. Of these 32 recruits, 18 (56%) ranked personal sense of right and wrong as most influential, while 10 (31%) ranked attitude first. This poses the problem of how to improve police reactions to repeated exposures to people whose attitudes result from what are often vulnerable and compromised states, requiring more research from the officer’s perspective [47]. In any case, these preliminary results suggest that police have strong beliefs about their use of discretion towards PWUD and what shapes it. Developing and validating instruments that capitalize on this knowledge would yield training and policies that better direct police discretion towards harm reduction, and effectively evaluate the measures when they are implemented.

The results also call for more nuanced ways to measure police stigma towards PWUD. Standard measures concern themselves with attitudes that affect social relations with PWUD or asses their inherent blameworthiness for their condition, but these are less relevant when we consider how stigma relates to the police role. That an overwhelming majority of officers characterized people with OUD as untruthful is an important insight relevant to the police role, and a useful instrument would measure this stigma specifically, such as the trustworthiness of PWUD as witnesses and complainants, and their perceived motives during encounters with police. We may also care about the extent to which they are perceived as being insincere in their statements and actions to avoid interrupting their consumption of drugs, the degree to which they will falsely portray themselves as motivated to enter treatment to avoid criminal charges, and if police perceive poor attitudes that disincentivize discretion are inherent to the demeanor of PWUD. This stigma is also pronounced among medical care providers, and its measurement benefits from dedicated instruments [22], suggesting the need for a reliable scale for measuring police stigma towards PWUD. To the extent police intentions are shaped by stigma, training about how the biology of addiction affects behavior, use of sympathetic narratives, and an emphasis on the structural and systemic causes of addiction may then mitigate such stigma [35] and moderate officers’ attitudes towards alternatives to arrest.

Syringe laws vary considerably between states, and officers report having less discretion when people possess them. For example, Missouri law distinguishes between unused syringes and those with drug residue in them (the latter being unlawful to possess), and all syringe possession for illicit use is a felony in Indiana (Code § 16–42-19–18 (2017)), likely contributing to officers’ reluctance to use discretion. Analyzed separately, the Indiana mean for control over confiscation was 3.79 as compared to 4.16 for the remainder of the sample, suggesting reforms that legalize syringe possession or categorize it as a less serious offense could increase police use of discretion. Although some observe that officers may be confiscating syringes as a public health measure to remove nonsterile ones from circulation, no study we are aware of has revealed this rationale among police, while the state-level status of syringes as contraband regardless of their sterility provides sufficient legal cause, and often a corresponding motivation, to confiscate them regardless of their sterility.

While policies that would emphasize health over criminalization are being contemplated in several jurisdictions, supervisors might in the meantime legitimize the health-oriented use of discretion among their officers. If further research confirms that supervisors are among the most influential groups in shaping police officers’ discretionary behavior, training and policies should specifically focus on supervisors’ knowledge, beliefs and attitudes about addiction, effective treatment, and its potential to reduce crime and future arrests. Research should explore the extent to which supervisors who explicitly accord discretionary prerogatives for drug enforcement to the officers enhance those officers’ perceptions of behavioral control. Reducing the perceived seriousness of an offense could also increase the normative acceptability of discretion. As laws shift and decriminalization becomes more widespread, research should determine whether these developments change officer perceptions about the seriousness of the wider class of drug-related offenses. Offenses such as illicit drug or syringe possession are nonviolent and usually misdemeanors, and police face a series of choices about how to respond.

The most notable limitations of this study derive from the sample of 259 police employees from 3 states and dozens of agencies. Geographic diversity leaves open the possibility of heterogeneity among respondents, although interstate comparisons suggest the effects were limited. All factors that influenced a decision to arrest were ranked identically between states except in one case where one state’s officers ranked peer influences higher than those of friends and family. That the data here is largely consistent across states suggests the insights here are generalizable enough to provide direction for future research.

Nonresponse bias cannot be ruled out as affecting the results, but a high rate of completions among starts in an environment where participants were asked to complete the survey by a live instructor with time set aside during class suggests a minority of participants never started the survey. Another limitation is that some officers who completed these instruments were employed by agencies that perceived value in training that emphasizes an alignment between improved health outcomes for PWUD and the occupational safety and wellness of police, while others self-selected for the training. The data here were collected before the training was conducted to avoid contamination of the sample, but some agencies already had programs in place that sent people with substance use disorder to treatment if they desired it. These factors present the possibility that police officers in these agencies have been conditioned to view drug enforcement through a discretionary lens, or that a social desirability bias influenced their responses. Overcoming these limitations requires building on the base of this study. Elicitation interviews in different jurisdictions and instrument development will enable researchers to test if the constructs of the TPB provide generalizable guidance on how to best shape and direct police discretionary behavior.

## Conclusion

The results of this study suggest respondents believe they have considerable discretion over the drug laws they enforce, the arrests they make, and the items they confiscate. They report using this discretion in different ways for different reasons, distinguishing between their intentions to confiscate contraband or to charge a person for possessing it. Decriminalizing drug and syringe possession would stop police from making arrests for those acts, but the laws that govern the risk and criminal behaviors associated with substance use disorder would remain in effect, and police would continue to have discretion in enforcing them.

The results indicate many police believe treatment medications for addiction are effective, support referring people to treatment in lieu of arrest, and that doing so would increase trust in the police and improve community relations. What remains to be built out is an evidence-based account of how and why police use their discretion in these matters, and how to shape their use of discretion to achieve the ends that would better address the nation’s overdose crisis. At present, we have instruments that measure whether police believe certain things about addiction, overdose, treatment and PWUD generally, but we lack a reliable means to understand how policies and interventions affect discretionary intentions. Future research should address this. One step would explicitly link training about treatment and harm reduction with an acknowledgment of the police power of discretion. Another would create policies and training that directs the use of discretion based on research into what factors shape the behavioral intentions of police.

## Data Availability

The data utilized in this study may be disclosed for research purposes by contacting the corresponding author.

## Abbreviations

DOTS: Drug Overdose Trust and Safety
IRB: Institutional review board
HIV: Human immunodeficiency virus
MAT: Medications for addiction treatment
MOUD: Medications for opioid use disorder
OUD: Opioid use disorder
PWUD: People who use drugs
SHIELD: Safety and Health Integration in Enforcing the Laws on Drugs
SUD: Substance use disorder
TPB: Theory of planned behavior

## Footnotes

1 The authors prefer the term medications for opioid use disorder (MOUD), but used the acronym MAT, explained as “Medications for Addiction Treatment” because many officers understood its implications and were more familiar with it than MOUD, which would give them pause as a neologism.
